# Correspondence

**Published:** 1883-02

**Authors:** 


### Correspondence.

Copenhagen, December, 1882.

*To the Editors of the Buffalo Medical and Surgical Journal.*

Sirs—According to the desire, expressed by the International Medical Congress at its 7th session in London, 1881, and in consequence of later discussions on this subject, it has been resolved, that the 8th session of the Congress shall be held in Copenhagen.

Especially in order to prevent collisions with other medical congresses, we beg to request that you will, already now, in your esteemed journal, draw attention to the fact that the 8th session of the International Medical Congress will take place in Copenhagen during the days from the 10th to the 16th of August, 1884.

We are, Sir, Your Faithful Servants,

P. L. PANUM, President of the Organizing Committee of the Congress.

C. LANGE, Secretary General.

